# Optimizing Remote Sensing Image Retrieval Through a Hybrid Methodology

**DOI:** 10.3390/jimaging11060179

**Published:** 2025-05-28

**Authors:** Sujata Alegavi, Raghvendra Sedamkar

**Affiliations:** 1 Internet of Things Department, Thakur College of Engineering and Technology, Mumbai 400101, Maharashtra, India; 2 Computer Engineering Department, Thakur College of Engineering and Technology, Mumbai 400101, Maharashtra, India

**Keywords:** hyperspectral images (HSIs), synthetic aperture radar images (SAR), Convolutional Neural Networks (CNNs), pretrained networks, classification, retrieval, hybrid network

## Abstract

The contemporary challenge in remote sensing lies in the precise retrieval of increasingly abundant and high-resolution remotely sensed images (RS image) stored in expansive data warehouses. The heightened spatial and spectral resolutions, coupled with accelerated image acquisition rates, necessitate advanced tools for effective data management, retrieval, and exploitation. The classification of large-sized images at the pixel level generates substantial data, escalating the workload and search space for similarity measurement. Semantic-based image retrieval remains an open problem due to limitations in current artificial intelligence techniques. Furthermore, on-board storage constraints compel the application of numerous compression algorithms to reduce storage space, intensifying the difficulty of retrieving substantial, sensitive, and target-specific data. This research proposes an innovative hybrid approach to enhance the retrieval of remotely sensed images. The approach leverages multilevel classification and multiscale feature extraction strategies to enhance performance. The retrieval system comprises two primary phases: database building and retrieval. Initially, the proposed Multiscale Multiangle Mean-shift with Breaking Ties (MSMA-MSBT) algorithm selects informative unlabeled samples for hyperspectral and synthetic aperture radar images through an active learning strategy. Addressing the scaling and rotation variations in image capture, a flexible and dynamic algorithm, modified Deep Image Registration using Dynamic Inlier (IRDI), is introduced for image registration. Given the complexity of remote sensing images, feature extraction occurs at two levels. Low-level features are extracted using the modified Multiscale Multiangle Completed Local Binary Pattern (MSMA-CLBP) algorithm to capture local contexture features, while high-level features are obtained through a hybrid CNN structure combining pretrained networks (Alexnet, Caffenet, VGG-S, VGG-M, VGG-F, VGG-VDD-16, VGG-VDD-19) and a fully connected dense network. Fusion of low- and high-level features facilitates final class distinction, with soft thresholding mitigating misclassification issues. A region-based similarity measurement enhances matching percentages. Results, evaluated on high-resolution remote sensing datasets, demonstrate the effectiveness of the proposed method, outperforming traditional algorithms with an average accuracy of 86.66%. The hybrid retrieval system exhibits substantial improvements in classification accuracy, similarity measurement, and computational efficiency compared to state-of-the-art scene classification and retrieval methods.

## 1. Introduction

Global change, driven by evolving Earth land cover and use, faces new challenges amid the information revolution. Climate shifts and population demands have altered the global land use/cover map. Advancements in satellite-based sensors enhance geospatial mapping accuracy. Multispectral/hyperspectral imagery captures spectral signatures of varied materials, enabling terrain feature identification. Classification, crucial for delineating similar features, uses clustering algorithms and statistical structures. Medium spatial resolution sensor images pose classification accuracy challenges, addressed by advanced computing techniques. Supervised classification utilizes trained samples, while unsupervised classification handles scenarios without training data, fostering improved accuracy in land cover mapping and monitoring.

### Remotely Sensed (RS) Image Processing

In the realm of remote sensing, HSI and SAR images are extensively utilized to exploit the wealth of discrete spectral information inherent in an image. The complex nature of HSI/SAR scenes, involving combined pixels, vast data volumes, and limited training samples, presents a formidable challenge necessitating precise recognition methods. Spatial domain classifiers like support vector machines (SVMs) and Multiscale Breaking Ties (MSBT) have historically been employed in the initial phases of HSI/SAR image analysis. However, recent advancements focus on leveraging spectral and temporal data for enhanced image discrimination.

HSI and SAR images play a pivotal role in geo-indexing, target monitoring, area mapping, and environmental monitoring. HSI, possessing two spatial dimensions and one spectral dimension, forms a 3D cube, offering comprehensive information about the physical characteristics of sensed objects. The availability of spatial–spectral information has led to the development of various applications for classification and retrieval, posing challenges due to the high dimensionality of the data.

High-resolution data, collected through sophisticated sensors in Earth observation programs, contributes to the wealth of remote sensing images. HSI/SAR images, with their numerous bands (e.g., 220 bands), provide rich spectral information, facilitating the differentiation of materials. Spectral signatures unique to each material enable effective classification. Advanced sensors capturing fine spatial resolution data exacerbate the dimensionality problem in the spectral domain. The increased spatial resolution allows for the identification of minute spatial structures within the imagery.

## 2. Literature Survey

In the early stages of this era, extensive research has been conducted on remote sensing image processing, providing a comprehensive overview of diverse methodologies employed for image processing and evaluation in the field. This section offers insights into the depth of research endeavors in this domain.

### 2.1. Preprocessing of HSI/SAR

Jaime Delgado et al. [1] proposed leveraging purist signatures in mixed pixels of hyperspectral images using spectro-unmixing to address spatial resolution limitations. Spatial preprocessing (SPP) techniques were employed before end member identification, but the associated cost and processing time raised concerns. G. Martín et al. [2] introduced three parallel graphic processing unit (GPU) implementations of the SPP algorithm, marking the first GPU application for spatial preprocessing. Their evaluation showcased reduced execution time, demonstrating real-time feasibility for Airborne Visible/Infrared Imaging Spectrometer (AVIRIS) (https://www.jpl.nasa.gov/missions/airborne-visible-infrared-imaging-spectrometer-aviris/) Accessed on 1 July 2021 sensor data collection.

### 2.2. Feature Extraction of HSI/SAR

L. Fang [3] introduced a novel Deep Hashing Neural Network (DHNN) model for HSI classification, employing a trained network to extract similarity-preserving features. The model incorporates a hashing layer to convert real-value features into binary ones, expediting distance computation. A customized loss function is designed to optimize feature distances in Hamming space, and the DHNN output is utilized by a support vector machine (SVM) for HSI classification.

Cui Binge et al. [4] proposed a Nonlinear Robust Principal Component Analysis (NRPCA)-based feature extraction method combined with Minimum Noise Fraction (MNF) to address challenges in hyperspectral image analysis. The approach involves reducing image size through average image fusion, applying NRPCA to each band, and utilizing MNF for low-level function feature extraction. The SVM method is then employed for identification, considering higher signal-to-noise ratios in the transformed results based on linear quality transformation and signal-to-noise ratio considerations.

### 2.3. Classification of Hyperspectral Images/Synthetic Aperture Radar Images

The limited availability of extensive training data poses a challenge for Deep Learning Neural Network (DLNN) classification in hyperspectral imaging. Addressing this, tailored frameworks for specific tasks or regularization phases with a finite number of samples can enhance generalization. The MugNet network simplifies DLNN architecture for improved performance with few training samples. Additionally, employing transfer learning proves effective in enhancing DLNN accuracy across various scenarios [5].

In hyperspectral image classification, some studies focus on Active Learning (AL) frameworks that generate regions to constrain sampling. Traditional AL heuristics often overlook spatial uncertainty and batch mode approaches may neglect spatial homogeneity. Zhaohui Xue introduces an Enhanced Uncertainty Measure (EUM) considering community knowledge, utilizing super pixels generated by simple linear iterative clustering (SLIC) to enhance sample diversity. Experimental results demonstrate significant classification precision improvement compared to conventional methods [6].

### 2.4. Deep Learning Methods of Classification

Researchers have explored various multiscale spatial features to propose spectral–spatial fusion methods, leveraging regulated CNN for hyperspectral image classification. They reconstruct the spectral function as a 2D image and apply guided filters to remove spatial functions, addressing finite training sampling challenges [7]. The presented spectral–spatial fusion techniques demonstrate significant performance improvements and precise allocation. Additionally, a study discusses integrating multiscale representation in CNN to enhance object identification, visual recognition, and time series classification, particularly for space IR-point objects. The proposed multiscale convolution neural network (MCNN) aims to efficiently capture both fast and slow variables in IR signature features, considering motion effects and noise resilience for artifact classification [8].

### 2.5. HSI/SAR Retrieval System

Yansheng Li et al. [9] introduced the deep-hazardous neural networks Scale-Invariant Deep Hybrid Convolutional Neural Networks (SIDHCNNs) optimized with Compressed Sensing—Low-rank and Sparse-Representation-based Structured Image Registration (CS-LSRSIR) constraints for invariant source learning, aiming for maximum capacity utilization. The proposed SIDHCNNs undergo quantitative evaluation using a dual-source remote sensing dataset comprising 8 standard terrain cover categories with 10 thousand dual samples for each category.

Osman Emre Dai et al. [10] presented a novel content-based image recovery method for remote sensing (RS), involving an image definition approach to distinguish spatial and spectral details. They employ a supervised recovery approach, utilizing descriptors of RS image content, and introduce three spectral descriptors. The method utilizes sparse reconstruction-based classifiers for single-label and multilabel RS image recuperation, enhancing recovery efficiency through improved sensitivity to different dictionary terms.

## 3. Implementation

**Figure 1:**
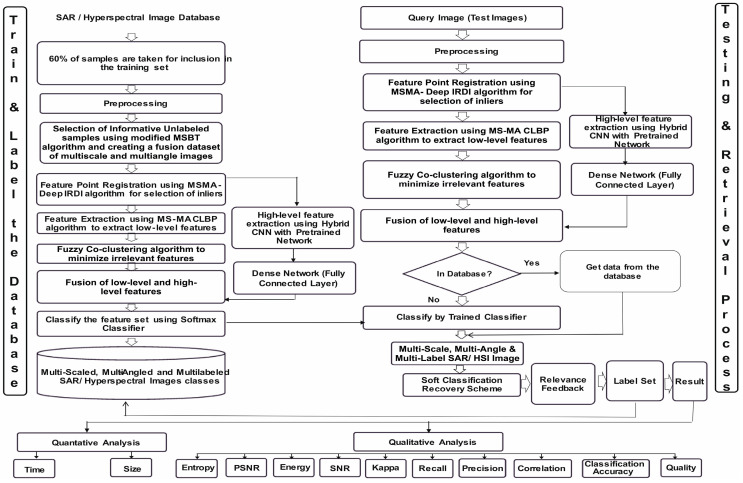
Proposed hybrid architecture for HSI/SAR image retrieval.

The depicted architecture in Figure 1 below, of the proposed HSI/SAR hybrid retrieval system exhibits two distinct modules: the “Database Building” section, responsible for training, labeling, and storing training samples, and the “Retrieval Process” section, focused on testing and retrieving query images. The recovery process for a given query image patch initiates with feature extraction, utilizing precalculated target patch features stored in the “building database” section. Leveraging these features, the semantic category of the query image patch is determined, and similarity with target category patches is computed. The retrieval results are subsequently presented in an ordered list. Preprocessing techniques are employed to enhance retrieval precision by mitigating image noise and reducing the corresponding database space. Below is the discussion of every component of the architecture in detail.

### 3.1. Train and Label Database (Database Building)

The primary aim of this architectural phase is to conduct training, labeling of selected samples, and establish a labeled database. This involves sequential stages of preprocessing, sample selection for the training set, image registration, feature extraction, classification, and database categorization. For the creation of a labeled dataset, 60% of diverse category samples are chosen as training samples for raw HSI/SAR images. These images undergo preprocessing using Gabor and independent component analysis (ICA) filters for noise removal and oriental feature capture. Employing scaling, rotation, and fusion, a Multiscale–Multiangle dataset (MSMA) of HSI/SAR images is generated.

To streamline sample selection, the MSMA-MSBT algorithm is introduced, focusing on efficient and informative unlabeled sample inclusion in the training set. Furthermore, feature point registration is performed using the modified DeepIRDI technique, enhancing the robustness of feature point enrollment. These inliers are then fed into the modified MSMA-CLBP module and the hybrid pretrained CNN classifier module. Low-level features are extracted using the modified MSMA-CLBP algorithm, addressing the efficiency gap in CNN for low-level feature capture in remote sensing images.

High-level features are obtained through a CNN structure, leveraging initial layers of pretrained networks such as Alexnet [11], Caffenet [12], VGG-F, VGG-M, VGG-S, VGG-VDD-16, and VGG-VDD-19 [13]. These features are subsequently input into the dense network to derive high-level features. Fuzzy co-clustering is applied to reduce irrelevant local features extracted by the modified MSMA-CLBP algorithm, addressing the curse of dimensionality in HSI/SAR images. The fuzzy co-clustering algorithm automatically calculates feature weights, optimizing data processing. The fused local features from the fuzzy co-clustering module and high-level features from the hybrid pretrained CNN module are input into the classifier, forming the proposed hybrid retrieval network. A SoftMax classifier is employed for final classification, generating multilabeled HSI/SAR image classes. To tackle post-classification challenges, particularly in mixed pixels prevalent in HSI/SAR images, a soft classification recovery process is proposed. This process adaptively adjusts threshold values for effective pixel assignment in regions of mixed pixels, addressing difficulties in class distinction through normal classification methods.

### 3.2. Testing and Retrieval Process (Retrieval Process)

This section is dedicated to querying the labeled database for the most suitable images based on a given query. Upon input of a query image, its features are automatically extracted following the database creation process, including preprocessing. Two scenarios are considered: whether the query image features exist in the designated image database or not. If the features are present, relevant information is directly retrieved from the database. In the absence of features, the query image undergoes classification by the trained hybrid classifier, and subsequent determination of similarities between the query and target images is conducted.

Considering the characteristics of HSI/SAR images, a region-based similarity measurement is introduced, utilizing patch similarity. To address potential classification errors, the query image is not only matched with the most relevant image in the database but also with other relevant images belonging to the same category. To mitigate the inevitable classification errors, a soft thresholding technique is proposed, derived from a confusion matrix. The utilization of various Compute Unified Device Architecture (CUDA) libraries and GPU processing is emphasized for enhanced speed and processing efficiency compared to central processing unit (CPU)-based operations.

### 3.3. Multiscale, Multiangle, and Multiscale–Multiangle (Fused) Dataset 

#### 3.3.1. Scaling

In the offline datasets, HSI/SAR images are first scaled at different levels to get the scaled dataset; then, these scaled images are rotated to get the angle dataset. Finally, these scaled and rotated images are fused together to form a fusion dataset. The different resolutions used for training and testing are 512 × 512, 256 × 256, and 64 × 64. These offline datasets of HSI/SAR images are further used for training and testing purpose. For scaling Gaussian pyramid decomposition is used in which the original HSI/SAR image is taken as HSI/SAR-1. Furthermore, this HSI/SAR-1 image is convolved with a Gaussian kernel and downsampled to get HSI/SAR-2 image. c refers to the different scales applied for generating multiscale images. The grey scale value in the HSI/SAR-1 layer is obtained by:

(1) $$HSI/\;SAR-1\;\;(i,\;j)=\sum_{m=-c}^{c}\sum_{n=-c}^{c}G\left(m,\;n\right)*I_{l-1}\;(2i-1-m,\;2j-1-n)$$

where * is the convolution operation*, HSI/SAR*−1∈{2, 3, …, l}, I_l−1_ represents the previous image reconstructed with Gaussian layer l−1, l represents the number of layers of Gaussian pyramid, and the Gaussian window is represented as follows,

(2) $$G\left(m,n\right)=\frac{1}{2\pi \sigma^{2}}e^{-\frac{\left(m^{2}+n^{2}\right)}{2\sigma^{2}}}$$

where σ refers to the variance that relates to the Gaussian filter and *HSI/SAR-1, HSI/SAR-*2…, *HSI/SAR-N* are obtained by continuous convolution and downsampling operation. Multiple images of 64 × 64, 256 × 256, and 512 × 512 which will yield multiscaled image dataset are selected.

#### 3.3.2. Rotating

To understand the effect of rotation on HSI/SAR images and to analyze the performance of various algorithms on such images, HSI/SAR images are rotated at various angles such as 900, 1800, and 2700, which yields multiangled database which are further used for training and testing purpose.

#### 3.3.3. Fused (MS-MA)

HSI/SAR images are captured using different sensors which have different resolutions and they are also captured with different instantaneous field of view, which distorts the image in scale and angle. To study this effect, scaled and rotated HSI/SAR images are fused together to form a database consisting of both the variations of scaled and rotated images. Different variations of fused datasets, such as 512 × 512-90°, 512 × 512-180°, 512 × 512-270°, 256 × 256-90°, 256 × 256-180°, 256 × 256-270°, 64 × 64-90°, 64 × 64-180°, and 64 × 64-270°, are formed. These images are further used for training and testing purposes. 

### 3.4. Proposed Modified MSMA-MSBT

The algorithm blends the Breaking Ties (BT) technique and mean shift (MS) cluster to choose the most explanatory training sampling. To more accurately perform the MS-MA CNN algorithm, we describe various variables. p provides the number of specimens for each iteration to be labeled. We have used an algorithm to construct a collection of N vectors. We take one part of N as NV training package with u basic training sampling and we take the NY package with unlabeled A-w samples to verify the other part of the N. NV is named after the training set, and stands as $\;B_{S}=\{(ai_{r},ci_{u}){\}}_{r=1}^{d}$. If the validation range is unmarked, NY = $\{ci_{u}{\}}_{r=1}^{A-w}$, we select a suggested algorithm, p new samples, such as ${\;D}_{MS-MA\;}=\{ci_{u}{\}}_{r=1}^{t}$ from the validated set, which we name $D_{MS-MA\;}=\{ai_{r},ci_{u}{\}}_{r=1}^{p}$ and then add $D_{MS-MA\;}$ into BT [14].

The parameter “t” represents the number of new samples that are selected by the MS-MA (Multiscale Multiangle) CNN algorithm from the validation set to be added to the current training set in each iteration.

#### 3.4.1. Algorithm Overview

##### Key Variables and Parameters

Variable “*p*” denotes the number of specimens labeled for each iteration.

The algorithm constructs a collection of N vectors using a specific technique.

NV represents the training set, composed of u basic training samples, while NY is the set for validation with unmarked A-w samples.

The algorithm selects *p* new samples from the validated set, denoted as ${\;D}_{MS-MA\;}=\{ci_{u}{\}}_{r=1}^{t}$ from the validated set, names them $D_{MS-MA\;}=\{ai_{r},ci_{u}{\}}_{r=1}^{p}$, and then adds $D_{MS-MA\;}$ into BT.

##### Algorithm Steps

A-w samples are used to verify the other part of the N. NV is named after the training set, and stands as$\;B_{S}=\{(ai_{r},ci_{u}){\}}_{r=1}^{d}$. If the validation range is unmarked, NY = $\{ci_{u}{\}}_{r=1}^{A-w}$, we select a suggested algorithm, *p* new samples, such as ${\;D}_{MS-MA\;}=\{ci_{u}{\}}_{r=1}^{t}$ from the validated set, which we name $D_{MS-MA\;}=\{ai_{r},ci_{u}{\}}_{r=1}^{p}$, and then we add $D_{MS-MA\;}$ into BT.

**Input:** Basic classifier parameter from multiscale, etraining set, BT = $(ai_{u},ci_{u}{)}_{r=1}^{d}\;$, validating set,$N_{y}=\{Xi_{u}{\}}_{r=1}^{A-w}$ mean-shift parameters, f, candidate samples parameter, β.

θ^, ê, and â, signify an estimated value, derived from the algorithm.

e, a, and θ refer to the actual values.

1. Train the MS-MA CNN classifier and receive the classifier parametere^.
2. Forecast and get NY samples using the Classifier(a^ir,ciu)r=1A−w.
3. For each sample in the subset (a^ir,cir)r=1A−w, compute its BT value as:
  (3) θ^ir=mint(a^ir=j|cir,e^)−minp(air=j|cir,e^)
4. Arrange {θ^}r=1A−w in ascending order as {θ^vr}r=1A−w, and depending on the multilabel $\{v_{r}{\}}_{r=1}^{A-w}$, take out the first β. *p* samples from NY become NBT = $\{N_{vr}{\}}_{r=1}^{\beta .p}$.
5. Find out the *p* set of samples for $N_{BT}=\{N_{vr}{\}}_{r=1}^{\beta .p}$ by mean shift parameter f. Choose a random sample for every sample subset and position all selected *p* sampling p$N_{MS-MA\;}$.
  $N_{MS-MA\;}=\{Ni_{g}{\}}_{g=1}^{p}Multi\;-$ Label $N_{MS-MA\;}$ to obtain,
(4)             NMS−MA ={(a^ig,cig)}g=1p
6. Update BT and NY.
  (5) $${\;\;\;\;\;\;\;\;\;\;\;\;\;\;\;B}_{T}=B_{T}\cup B_{MS-MA\;};N_{y}=N_{y}-N_{MS-MA\;}$$
7. Refer to step (1) if a condition is not met; otherwise, prevent repetition.
8. Initialization of multiscale, multiangle CNN for semantic segmentation: setting the size of three image patches as 512 × 512, 128 × 128, and 64 × 64 and image angles as 90°, 180°, and 270°.
9. Fused dataset is obtained by combining resolution and angle datasets. That is, integrating angle and resolution which is 90°, 180°, 270° and 512 × 512, 128 × 128, 64 × 64 resolution.
10. Extract patches in three dimensions and angles, and normalize image patches.
11. Branch the image patches collected as research specimens and train samples.
12. Train network using the decent gradient algorithm in an end-to-end way.
13. Usage of the trained network to achieve labels for the test samples.

### 3.5. Inliers and Outliers in Feature Matching

#### 3.5.1. Understanding Inliers and Outliers in Feature Matching

When we compare two images—whether they come from satellites, drones, or any other remote sensing source—we rely on feature matching to align them correctly. In this process, the terms inliers and outliers become very important.

- Inliers are the points in an image that correctly match between the two images. They follow the actual transformation (like rotation, scaling, or translation) between the images, ensuring accurate alignment.
- Outliers, on the other hand, are incorrect matches. These could be caused by noise, differences in lighting, or distortions in the images. Too many outliers can lead to errors in the final image alignment.

#### 3.5.2. Challenges in Feature Matching

1. **Sensor Noise**

Different sensors capture images in different ways. Hyperspectral images may be affected by atmospheric noise, while SAR images often suffer from speckle noise, making it harder to detect correct feature points.

2. **Geometric Distortions**

When two images are taken from different angles or sensors, they may appear distorted. SAR images, for example, often have a foreshortening effect, which changes the apparent shape of objects.

3. **Errors in Image Alignment**

Traditional methods (like RANSAC [15]) rely on fixed rules to filter inliers and outliers, but these do not always work well for all types of images. If the filtering process is not adaptive, the method may struggle with different image conditions.

Our proposed modified DeepIRDI algorithm improves feature matching by making the process smarter and more adaptive by:

1. **Better Selection of Inliers**

Instead of using a strict rule to decide which matches are correct, our approach assigns a probability score to each match. This means the system can adjust dynamically, depending on the quality of the image.

2. **Handles Images at Different Scales**

Some features may appear bigger or smaller in different images. Our approach works at multiple scales, ensuring that important details are captured correctly even when images differ in size or resolution.

3. **Deals with Geometric Distortions**

To prevent errors in alignment, our method checks the spatial consistency of feature points. If a match does not fit well within the overall transformation of the image, it is more likely to be discarded as an outlier.

4. **Faster and More Efficient**

Using GPU acceleration, our method processes images much faster, making it suitable for handling large datasets, such as those used in land cover mapping and disaster monitoring.

### 3.6. Proposed Modified DeepIRDI Algorithm

In the process of inlier selection denoted as M × N, a probability matrix PR is generated. This matrix is then subjected to a transformation solver based on the Gaussian mixture model (GMM). The entry PR [m, n] in this matrix represents the probability of matching *x_n_* and *y_m_*. Assuming that *x_n_* corresponds to *y_m_*, a higher probability is reflected in a larger PR [m; n]. This elevated probability indicates a significant correspondence between *x_n_* and *y_m_*, allowing for a visible alignment of characteristics in the respective pair.

The determination of probabilities involves the utilization of both convolution features and geometric structural information. The PR matrix is obtained through a defined operation [16].

Prepare the M×N convolutional cost matrix $\;c_{\theta }^{conv}$ by

(6) $$c_{\theta }^{conv}\left[m,\;n\right]=\left\{\begin{array}{c} \frac{d\left(y_{m},x_{n}\right)}{d_{\theta }^{max}},condition\;1\\ 1,\;\;\;otherwise \end{array}\right.$$

where *x_n_* is the point at index *n* in point set X and *y_m_* is the point at index m in point set Y, and$\;d_{\theta }^{max}$ is the maximum distance of all matched feature points pairs under threshold ϴ.

Equation (6), shows how to extract and match features of HSI/SAR images and detect objects in a different location captured with a different angle and a different viewpoint. $c^{geo}$ is acquired by performing a χ 2 test. Both $\;c_{\theta }^{conv}$ and $c^{geo}$ are valued in [0, 1]. The following equations elaborate on how the geometric transformation is estimated, where ${\;h}_{m}^{y}$(b) and ${\;h}_{n}^{x}$ (b) denotes the number of points decreased in the b^th^ bin surrounding y_m_ and x_n_, respectively.

(7) $$c^{geo}[m,n]=\frac{1}{2}\sum_{b=1}^{B}\frac{[h_{m}^{y}(b)-h_{n}^{x}(b){]}^{2}}{h_{m}^{y}(b)+h_{n}^{x}(b)}$$

Using an element-wise Hadamard product, we calculate an integrated array matrix C (denoted by⊙):

(8) $$C=C_{\theta }^{conv}⊙C^{geo}$$

Finally, the prior probability matrix is calculated using:

(9) $$P_{R}[m,\;n]=\left\{\begin{array}{c} 1,if\;y_{m}\;and\;x_{n}\;are\;corresponding\\ \frac{1-\in }{N},otherwise. \end{array}\right.\;\in is hyperparameter valued in [0 and 1])$$

Furthermore, optimal parameters of transformation like (W, σ^2^, ω) are used. The aim of such an approach is to maximize a likelihood function or decrease the negative log-likelihood function equitably: pold (m|x_n_) denotes a posterior likelihood. The equation may be rewritten after expanding this equation and omitting derivative redundant terms as:

(10) $$Q\left(W,\sigma^{2},\omega \right)=\frac{1}{2\sigma^{2}}\sum_{n=1}^{N}.\sum_{m=1}^{M}pold\left(m|x_{n}\right)||x_{n}-\tau (y_{m},W)|{|}^{2}\;-\frac{1}{2}N_{p}log\frac{\left(\alpha^{2}\omega \right)}{\left(1-\omega \right)}-Nlog\left(\omega \right)$$

where W is the transformation parameter andω is the weighting parameter.

The detailed implementation of the Modified Deep IRDI Algorithm is shown in Algorithm 1.

**Algorithm 1.** Modified Deep IRDI Algorithm Implementation.**Input**: A and B are the two images where ‘A’ is the reference image and ‘B’ is the captured image.
1.Initialize parameters from multiscale and multiangle HSI/SAR images.
2.Prematch and choose the multilabel feature point sets *F* and E from A and *B.*
3.Initialize ρ=ρ^.
4.**do while**5.               **for each *i* iterations:**
6.                     Calculate the array matrix for convolutional features                     $c_{\theta }^{conv}$as per Equation (6).
7.                     Calculate the array matrix for the geometric structure                     $c^{geo}$as per Equation (7).
8.                     Calculate the array matrix


(11)
$$C=C_{\theta }^{conv}⊙C^{geo}$$


                         Solve the array matrix C linear assignment.
9.                     Calculate the prior probability matrix *P_R_* using Equation (9).10.                   Update the threshold ρ ← ρ– α.11.            **end**12.**E-Steps:**


(12)
$$P_{0}\left[m,\;n\right]=\frac{P_{R}\left[m,\;n\right]g_{m}\left(x_{n}\right)}{P\left(x_{n}\right)}$$


13.Compute posterior probability matrix *P_O_* by14.  **end**15.  While there is no convergence of equation 10.16.  Calculate the transformed picture IZ using interpolation with a thin-plate spline.17.  **Output: IZ**


### 3.7. Proposed Modified MSMA-CLBP Algorithm

The modified MSMA-CLBP algorithm is proposed for low-level feature extraction, to improve the efficiency of the system. In the proposed system, the HSI/SAR image dataset is taken as input. For preprocessing, various filters like the Gabor filter and ICA filter are used for removing the noise from the image. Preprocessing not only clears data of noise but also uniformly modifies the data to be accepted by the network. The modified MSMA-CLBP algorithm is used for low-level feature extraction. Spatial features as well as Speeded-Up Robust Features (SURF) are extracted. Exhaustive feature matches feature set 1 to the nearest neighbors in feature set 2 by computing the pair-wise distance between feature vectors. In the next step, all results are fused according to the variance [17].

**Require: Preparation of training dataset and testing dataset**

1: Capture Gabor operator’s characteristics by converging input images.

2: Adjust the MS-CLBP operator parameters (m, r) and pick the optimal criterion. 

3: Achieve MS-CLBP characteristics depending on the Gabor feature space for every image. 

4: Build the low-level features by the MS-CLBP characteristics.

Assure: Works at the lowest level

The Gabor function consists of two parts: 1. a real part and 2. an imaginary part. By filtering, the real part, the image, and the imaginary part is used mainly for edge detection. The mathematical expression is as follows:

(13) $$g\left(x,y;\lambda ,\;\theta ,\sigma ,\gamma \right)=\exp\left(-\frac{x_{0}^{2}+\gamma^{2}\;y_{0}^{2}}{2\sigma^{2}}\right)\;.\exp(i(2\pi \frac{x_{0}}{\lambda }+\varphi ))$$

where:

$$\{x_{0}\;=\;\mathrm{xcos}ϴ\;+\;\mathrm{ysin}ϴ$$

$$y_{0}\;=\;-\mathrm{xsin}ϴ\;+\;\mathrm{ycos}ϴ\}$$

where x and y are the region of the pixels in the area. The LBP is utilized as a local texture characteristics description. The center pixel Z LBP value is described as:

(14) $$LBPq,e\left(Z\right)=s\left(\;g_{i\;}-g_{c}\right)2^{i}$$

where e is the length from the center point Z, q is the number of adjacent neighbors, gi is the neighbor’s grey value, and x = ($g_{i\;}-g_{c}$) is the variation among the center pixel and every neighbor. The proposed CLBP algorithm is based on an LBP algorithm.

(15) $$\;CLBP_{M}m,r=\sum_{i=0}^{m-1}s\left(D_{i}-T\right)$$

(16) $$2^{i}CLBP_{S}m,r=\sum_{i=0}^{m-1}s\left(\;g_{i\;}-g_{c}\right)2^{i}$$

(17) $$CLBP_{C}m,r=s\left(g_{c}-g_{n\;}\right)$$

$$where\;D_{i}=g_{i}-g_{c},g_{N}=\frac{1}{N}\overset{N-1}{\underset{j=0}{\sum g_{j}}},T=\frac{1}{N}\sum_{j=0}^{N-1}\frac{1}{M}\sum_{i=0}^{m-1}\left(g_{i}-g_{c}\right)$$

where gn helps with capturing fine-grained texture variations around a pixel and gN helps with capturing the overall brightness contrast of a pixel against its neighborhood.

One downside of the CLBP algorithm is that it defines only native single-scale and oriented characteristics. Based on the CLBP, the modified CLBP algorithm is proposed.

## 4. Results and Discussions

The experiments were conducted on a MATLAB 2018b platform, utilizing a system with an X64-based PC, Intel Core i5-7400 CPU @ 3.00 GHz, 8 GB RAM, NVIDIA GeForce GTX 1050 Ti GPU, and Windows 10 Pro OS. The proposed hybrid retrieval system underwent testing using both offline and online datasets. The offline dataset, comprising four classes with 100 images each, was meticulously prepared by applying scaling, rotation, and fusion to instill diversity for robust network training. Thirty distinct datasets, totaling over 10,000 images across different categories, were employed for training and testing. Benchmark hyperspectral datasets, including AID, UCMerced Landuse, WHU-RS19, Botswana, and others, were incorporated. The training set was constructed using 60% of images from various categories. Qualitative and quantitative experiments affirmed the effectiveness of the proposed hybrid retrieval model, demonstrating superior performance compared to other state-of-the-art algorithms.

### 4.1. Results of Proposed MSMA-MSBT Algorithm for Online Datasets

The AID dataset results are tabulated, comprising 10,000 images. The MSMA-MSBT algorithm, proposed for training set enrichment, selects 7090 highly informative unlabeled samples based on uncertainty, leading to increased feature diversity. The selection of samples varies across datasets, contingent on the level of uncertainty associated with each sample. The AID dataset results depicted in Table 1 showcase the selection of 7090 highly informative, uncertain, and unlabeled samples out of a total of 10,000 images by the MSMA-MSBT algorithm. This approach emphasizes selecting samples with more uncertainty to enhance feature diversity. The method involves choosing the cluster mean and adding the most uncertain samples to the training set, thereby strengthening the classification model. With 30 different classes in the AID dataset, the selection process prioritizes classes with distinctive features. Notably, more samples are chosen from classes like Dense Residential, contributing to efficient classifier training by reducing dimensionality.

#### Comparison of Performance of MSMA-MSBT with State-of-the-Art Algorithms

The proposed MSMA-MSBT method is compared with the SIFT algorithm using satellite and UAV datasets as shown in Table 2.

The table clearly illustrates SIFT’s poor performance, particularly in the case of SAR images captured by UAV. The multitemporal nature of these images, along with diverse sensors, introduces atmospheric noise, significantly impacting the images. Lower-altitude captures amplify the influence of atmospheric noise, making clear categorization and sample selection challenging. While results for satellite-captured images are comparable, the proposed method consistently outperforms the SIFT-based technique in terms of sample selection and classification accuracy. The classification accuracy for both satellite and UAV datasets is superior when employing the proposed method as opposed to the SIFT method. The selection of highly informative and unlabeled samples contributes to the effective training of the classifier using the proposed technique.

### 4.2. Results of Proposed Modified DeepIRDI Algorithm for Online Datasets

#### 4.2.1. Phase I and II: Training and Testing Phase

**Figure 2:**
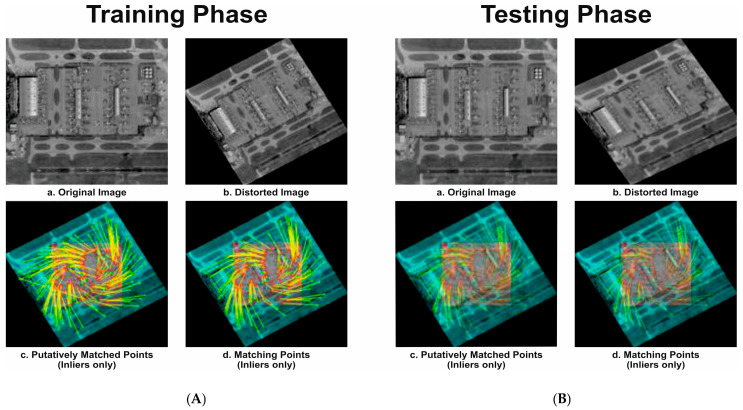
(**A**) Training and (**B**) testing of HSI/SAR image registration using the proposed modified DeepIRDI algorithm for AID/SAR dataset.

In Figure 2A, the feature registration in this stage utilizes the proposed modified DeepIRDI technique for enhanced matching of selected inliers. In this figure, ‘a’ represents the original image selected after preprocessing and sample selection for training using the proposed MSMA-MSBT technique. Figure ‘b’ shows a rotated and scaled image, common in HSI/SAR images captured by different sensors with varying instantaneous field of view (IFOV). Figure ‘c’ depicts all inliers and outliers, with inliers representing correctly displaced feature points and outliers being incorrectly mapped points in the captured image compared to the reference image. Figure ‘d’ showcases matching feature points using only inliers, resulting in a robust transformation parameter that aligns the orientation of the captured image with the reference image.

In Figure 2B, ‘a’ represents the satellite-captured image used as the query image for registration. The feature registration in this stage employs the proposed modified DeepIRDI technique for improved matching of selected inliers. Figure ‘b’ presents a distorted HSI/SAR image, common when captured by various sensors with different IFOVs. Figure ‘c’ illustrates all inliers and outliers, with inliers being correctly displaced feature points and outliers representing inaccurately mapped points in the captured image compared to the reference image. Figure ‘d’ exhibits matching feature points using only inliers, resulting in a robust transformation parameter that aligns the orientation of the captured image with the reference image.

#### 4.2.2. Comparison of Performance of Modified Deep IRDI with State-of-the-Art Algorithms

Table 3 presents a comparison between the proposed modified DeepIRDI algorithm and existing state-of-the-art algorithms for online datasets, specifically satellite and UAV datasets. Experimental results demonstrate that the proposed method consistently outperforms the existing algorithms. For satellite images, the proposed modified DeepIRDI achieves the highest accuracy of 98.65%, utilizing inliers for feature point registration. In contrast, RANSAC achieves 84.14%, using both inliers and outliers for registration. The simple image registration (IR) algorithm attains an accuracy of 95.65%, while SIFT lags behind at 71.71%. In the case of UAV images, the proposed method achieves an accuracy of 95.43%, outperforming RANSAC (79.25%), IR (93.37%), and SIFT (42.94%). The dynamic selection of inlier feature points contributes to the superior accuracy of the proposed modified DeepIRDI algorithm, establishing it as the most accurate among the compared state-of-the-art algorithms. The average accuracy falls within the range of 80% to 87%. The proposed technique exhibits superior performance, enhancing the overall accuracy of the system. The existing IR system achieves an accuracy of 95.65%, while the proposed modified DeepIRDI attains 98.65%, indicating a 4% increase in accuracy. The dynamic updating of the ‘k’ value in the algorithm enables a greater alignment of inliers compared to other techniques, fostering coherence between the captured image and the reference image, ultimately leading to improved image registration accuracy. 

The goal of this comparison is to evaluate the effectiveness, flexibility, and robustness of the proposed hybrid framework under varied image acquisition conditions. The figure highlights that existing algorithms tend to perform reasonably well on satellite data but often struggle with UAV-acquired datasets. This discrepancy is due to several factors. Satellite data generally offer broader, more stable coverage and consistent acquisition angles, whereas UAVs introduce challenges like variations in altitude, changes in lighting, more intricate textures, and smaller, highly dynamic scenes. These complexities introduce noise and inconsistencies that many conventional algorithms cannot efficiently handle.

The proposed method, however, demonstrates a superior and more balanced performance across both satellite and UAV images. This suggests a few critical strengths of the technique:

1. Cross-platform generalization: The proposed technique maintains high accuracy and classification reliability, regardless of whether the data source is a satellite or a UAV. This reflects its strong generalization capability.
2. Enhanced feature learning: The two-level feature extraction mechanism—combining handcrafted (modified MSMA-CLBP) and deep features (from pretrained CNNs)—provides a rich, hierarchical understanding of remote sensing scenes. The integration of these feature types helps to preserve both local textures and global semantic structures.
3. Mixed pixel handling: One of the most impressive results is the method’s resilience in handling mixed pixels, which are common in HSI and SAR images. UAV datasets, in particular, contain many such mixed pixels due to high scene complexity. The soft classification recovery strategy employed by the model enables finer pixel-wise decision making, leading to better classification results in these areas.
4. Consistent performance improvement: The performance gap between the proposed method and traditional approaches widens in UAV datasets, reinforcing the idea that the proposed technique is more robust under challenging conditions. This means the method does not just outperform others in controlled conditions but also excels in more realistic and variable environments.
5. Real-world applicability: The consistent and reliable performance on both satellite and UAV images positions this method as a highly viable solution for real-world applications like urban planning, agriculture monitoring, disaster management, and environmental studies, where data may come from multiple platforms and under varied conditions.

Published results are available in [20].

#### 4.2.3. Results of Proposed Modified DeepIRDI Algorithm for Offline (Multiscale (MS), Multiangle (MA), and Multiscale–Multiangle (MS-MA), Also Known as Fused) Datasets

Table 4 provides a detailed accuracy analysis for the proposed method compared to existing image registration algorithms, showcasing minimum, maximum, and average accuracy metrics with respect to an offline dataset. The average accuracy ranges from 80% to 87%, with a maximum accuracy of 98.65% observed in the angle variant dataset and a minimum accuracy of 77.25% in the same dataset using the proposed modified DeepIRDI technique. A comparison with the RANSAC algorithm, commonly used for HSI/SAR image registration, reveals average results ranging from 80% to 83%, with a maximum accuracy of 90.81% in the fusion dataset and a minimum accuracy of 73.83% in the same dataset. The proposed algorithm consistently outperforms the existing method, exhibiting an average 4% increase in accuracy attributed to its dynamic inlier selection technique.

### 4.3. Results of Training and Testing of Multiscale Multiangle Model for SAR/Hyperspectral Images on Various Pretrained Network for High-Level Feature Extraction

Table 5 displays experimental results on SAR/hyperspectral datasets using the proposed approach. VGG-S exhibits the highest accuracy among different pretrained CNN models, outperforming others. The models VGG-M and VGG-VDD-16 show similar results, while Caffenet, Alexnet, and VGG-VDD-19 achieve the same average accuracy, and VGG-F performs the least among these models. These comparisons are based on both original and multiscaled images. For scene classification, seven pretrained networks (Alexnet, Caffenet, VGG-S, VGG-M, VGG-F, VGG-VDD-16, and VGG-VDD-19) are utilized, fine-tuning them in the proposed approach. The analysis reveals that multiscale images outperform original-scale images, as they provide more informative features for class distinction. Among the pretrained models, VGG-S stands out, achieving higher accuracy due to specific characteristics in its convolutional layers. The proposed method attains accuracies around 97% for multiscaled images across all considered pretrained models.

Table 6 presents a comparison of the time required for training the network using GPU and CPU. The results indicate a significant reduction in the time per epoch when utilizing a dedicated GPU, owing to parallel processing capabilities, minimizing both time and computational complexity. The construction of mid-level features, convolutional layer count, and the number of epochs influence the training time. Fine-tuning HSI/SAR data in the initial layers of pretrained networks highlights the impact of different networks on varying data amounts. For a limited dataset, pretrained networks outperform new CNNs built from scratch, but with a larger dataset, constructing a new CNN becomes a viable option. The dedicated use of GPU substantially reduces computational costs.

### 4.4. Comparison of Classification Performance of the Traditional Methods with the Deep Learning Methods Including Our Proposed MSMA-CLBP CNN Method

Table 7 illustrates a comparison between traditional methods, deep learning models, and the proposed MSMA-CNN built from scratch. When a substantial number of training samples are available, building a CNN model from scratch is recommended, allowing for a dedicated design tailored to the input images. The graph clearly demonstrates that the proposed hybrid MSMA-CNN outperforms other traditional and deep learning techniques, achieving a 1% convergence. In comparison to traditional classification algorithms, MSMA-CNN excels in capturing deeper features, resulting in a nearly accurate classification map. The inherent structure of the CNN contributes to a significant increase in classification accuracy compared to existing traditional algorithms. 

Detailed results can be found in [24,25,26].

### 4.5. Comparison of Classification Performance of the Proposed Hybrid System with State-of-the-Art Algorithms for Online Datasets

Table 8 illustrates a comparison between the proposed multiscale pretrained CNN classification technique and existing state-of-the-art algorithms for online datasets. The results indicate a clear superiority of multiscale pretrained CNN networks, showing an overall 3% increase in average accuracy compared to traditional algorithms. The advantage lies in the initial training of pretrained networks with millions of parameters and fine-tuning the input images accordingly. Additionally, the consideration of multiscale characteristics allows for the extraction of more precise features, contributing to increased overall efficiency. Among the models, Multiscale VGG-S stands out, attributed to its smaller filter size and varied pooling layers, outperforming both traditional and other pretrained networks. In scenarios with a limited number of training samples, pretrained models demonstrate superior performance over traditional classification algorithms.

### 4.6. Comparison of Performance Parameters of Proposed Hybrid Architecture for HSI/SAR Image Retrieval on Offline Datasets

Table 9 presents a comprehensive set of performance metrics for evaluating the proposed hybrid retrieval system on offline datasets of hyperspectral imaging (HSI) and synthetic aperture radar (SAR) images. The table includes various image configurations, such as different rotation angles (0°, 90°, 180°, 270°), resolutions (64 × 64, 256 × 256, 512 × 512), and fused datasets that combine both rotation and scaling. The evaluation metrics used are both quantitative (Peak Signal-to-Noise Ratio (PSNR), Mean Squared Error (MSE), Root Mean Squared Error (RMSE), Mean Absolute Error (MAE), Signal to Noise Ratio (SNR)) and qualitative (Entropy, Energy, Correlation, Quality Index, Kappa coefficient).

The entropy values, ranging from 6.79 to 7.65, represent the amount of information or detail in the images. Higher entropy values, such as 7.65 for 270°-512 × 512, indicate richer information content. Energy, indicative of pixel intensity uniformity, peaks at 3.02 for 180°-512 × 512, suggesting strong textural consistency in that configuration. Correlation values measure texture similarity between the reference and processed image, with most configurations achieving values around 0.73–0.80, the highest being 0.80 in configurations like 90°-512 × 512, 180°-64 × 64, and 270°-512 × 512.

In terms of PSNR, which assesses image fidelity, the highest value is 16.02 for 90°-64 × 64, indicating excellent image quality. Complementary to this, MSE and RMSE are minimized in configurations like 0° and 90°, pointing to better reconstruction accuracy. Specifically, 0° has the lowest MSE (11.01) and RMSE (4.00), making it highly effective for preserving image detail during retrieval.

The Quality Index, representing structural similarity, increases in fused datasets. It peaks at 0.70 in configurations such as 180°-256 × 256, 180°-512 × 512, 270°-256 × 256, and 270°-512 × 512, demonstrating the advantage of multiresolution and multiangle data fusion in capturing structural integrity. Kappa coefficients, which measure inter-rater reliability and classification consistency, remain high (0.74 to 1.00) across most configurations, indicating stable and reliable classification results.

Signal-to-Noise Ratio (SNR) values, which reflect the strength of the image signal relative to background noise, lie between 7.11 and 7.87. Notably, 64 × 64 achieves the highest SNR (7.87), indicating clearer signal distinction. Lastly, Mean Absolute Error (MAE)—a measure of the average deviation between the predicted and actual values—is lowest in 90°-64 × 64 (7.06), underscoring this configuration’s effectiveness in precise feature reconstruction.

Table 9 demonstrates that incorporating multiscale and multiangle image variants significantly improves retrieval performance. Configurations such as 90°-64 × 64, 270°-512 × 512, and 180°-256 × 256 consistently show superior performance across multiple parameters, validating the efficacy of the proposed hybrid retrieval architecture in handling complex HSI/SAR datasets.

### 4.7. Comparison of Performance Parameters of Proposed Hybrid Architecture for HSI/SAR Image Retrieval on Online AID Dataset

Table 10 provides a detailed evaluation of the proposed hybrid retrieval system using the AID (Aerial Image Dataset), which comprises approximately 10,000 images across 30 diverse classes, offering a rich benchmark for assessing both quantitative and qualitative parameters. The average values for each metric are computed across all classes to ensure consistency in performance analysis. Entropy values, reflecting image complexity, range from 4.30 to 7.57 with an average of 6.70, while energy values, indicating texture uniformity, vary from 1.24 to 9.33 with a mean of 3.68, favoring configurations with smoother spatial patterns. Correlation values remain high, between 0.88 and 1.00 (average 0.98), signifying strong similarity between query and reference image patches, which is critical in remote sensing. PSNR values, indicative of image reconstruction quality, span 17.64 to 18.91 dB (average 18.29), and are inversely related to MSE, which ranges from 8.42 to 18.53 (average 10.05), suggesting efficient image fidelity. RMSE values are consistently low, between 2.90 and 3.36 (average 3.12), highlighting minimal pixel-level error. The Quality Index varies widely from 0.09 to 3.33, averaging at 0.37, integrating luminance, contrast, and correlation into a single structural similarity score. Kappa coefficients, assessing classification agreement, range from 0.39 to 0.99 with an average of 0.76, indicating strong reliability across classes. SNR values (5.19 to 6.46, average 5.82) suggest the systems effectiveness in preserving signal clarity amid noise, while MAE values (6.30 to 6.46, average 6.39) confirm low average deviation between predicted and original image values. Collectively, the table demonstrates that the proposed hybrid approach maintains high retrieval accuracy, consistent structural similarity, and reliable classification performance across a variety of remote sensing image classes.

### 4.8. Comparison of Performance of Region-Based Similarity Measure Algorithm with State-of-the-Art Algorithms

**Figure 3:**
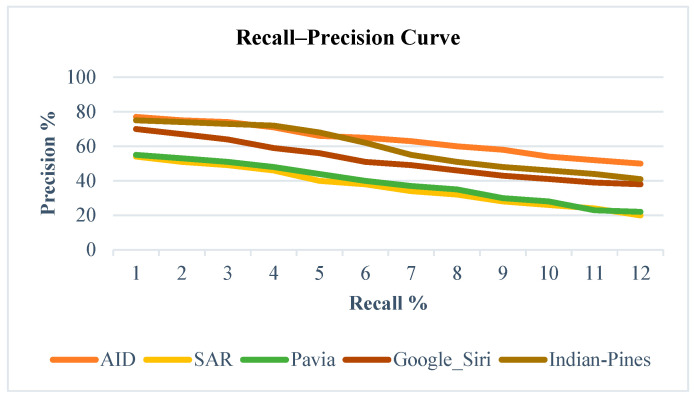
Comparison of recall–precision curve of the proposed technique for different datasets.

The graph in Figure 3 depicts the recall–precision curve generated for various datasets using the proposed hybrid retrieval technique. Notably, positive results are considered only when the outcome belongs to the same semantic category as the query. The statistical analysis is conducted on 300 images across 30 distinct categories, with the first 100 retrieved image patches taken into account. SAR and Pavia images exhibit suboptimal performance, primarily due to skew noise, which, even after denoising, diminishes essential features. Conversely, AID and Indian Pines outperform others, as high-resolution hyperspectral images are less affected by environmental noise, enabling effective global region similarity measurements. Detailed results are available in [34]. 

### 4.9. Comparison of Precision, Recall and F1-Score for AID, UC_MERCED and WHU_RS Datasets

Table 11 presents a comparison of precision values between existing methods and our proposed hybrid retrieval algorithm across various online datasets. Precision, in the context of HSI/SAR images, is determined by the relevance of retrieved images compared to the overall retrieved set using the hybrid retrieval approach. The existing precision is calculated based on the IRM algorithm for image retrieval. Higher precision values indicate a greater number of relevant images retrieved relative to irrelevant ones. Notably, in the case of the AID dataset, our proposed hybrid retrieval algorithm demonstrates enhanced precision.

The IRM method, relying on patch-based image segmentation followed by SVM classification, struggles to effectively capture features in remote sensed images, especially in areas with mixed regions falling into multiple classes. In contrast, our proposed method utilizes multilabeling, allowing a single patch to be associated with multiple classes, leading to more efficient retrieval and superior precision values. The precision values obtained with our proposed method consistently hover around 0.80 for all three challenging online datasets, outperforming IRM, which yields precision values ranging from 0.30 to 0.69.

The Table 12 presents a contrast between existing recall values and those achieved by our proposed hybrid retrieval algorithm across different online datasets.

For HSI/SAR images, the recall is determined by the portion of the total number of relevant instances effectively retrieved through the hybrid retrieval approach, while the existing recall is derived from the IRM algorithm for image retrieval. A higher recall value indicates that a significant proportion of relevant images is successfully retrieved in comparison to irrelevant ones. The IRM method relies on patch-based image segmentation followed by SVM classification, which falls short in effectively capturing the features of remote sensed images. Given that remote sensed images often contain mixed regions concentrated in multiple classes, making clear-cut classification challenging, our proposed method utilizes multilabeling. This approach allows a single patch to be associated with multiple classes, resulting in a more efficient retrieval outcome, and consequently, superior recall values. The recall values achieved using the proposed method consistently hover around 0.80 across all three challenging online datasets, surpassing the results obtained through IRM, which range between 0.33 to 0.66.

Precision and recall are quantitative and qualitative measures, respectively. To comprehensively assess the algorithm’s performance, the F1 score is considered, which combines precision and recall using a mathematical model to gauge the overall accuracy. A confusion matrix with low false positives and false negatives contributes to a higher F1 score. The Table 13 provides a comparison of existing F1 score values with our proposed hybrid retrieval algorithm for various online datasets.

The F1 score for HSI/SAR images is calculated based on precision and recall values obtained through the hybrid retrieval approach, while the existing F1 score is determined using precision and recall values from the IRM algorithm for image retrieval. Achieving low false positives and false negatives in retrieval results leads to a higher F1 score, indicating the system’s accurate retrieval of queried images. The IRM method relies on patch-based image segmentation followed by SVM classification, which struggles to effectively capture the features of remote sensed images. Due to the higher concentration of mixed regions in remote sensed images—areas that can fall into multiple classes and cannot be distinctly classified into one—the proposed method incorporates multilabeling. This approach allows a single patch to be assigned to multiple classes, resulting in a more efficient retrieval outcome and yielding improved precision, recall, and, subsequently, F1 score values. The F1 score values using the proposed method are around 0.80 for all three challenging online datasets, surpassing the results obtained using IRM, which provides F1 score values between 0.37 and 0.64.

### 4.10. Comparison of Retrieval Performance of the Hybrid System with State-of-the-Art Algorithms

**Figure 4:**
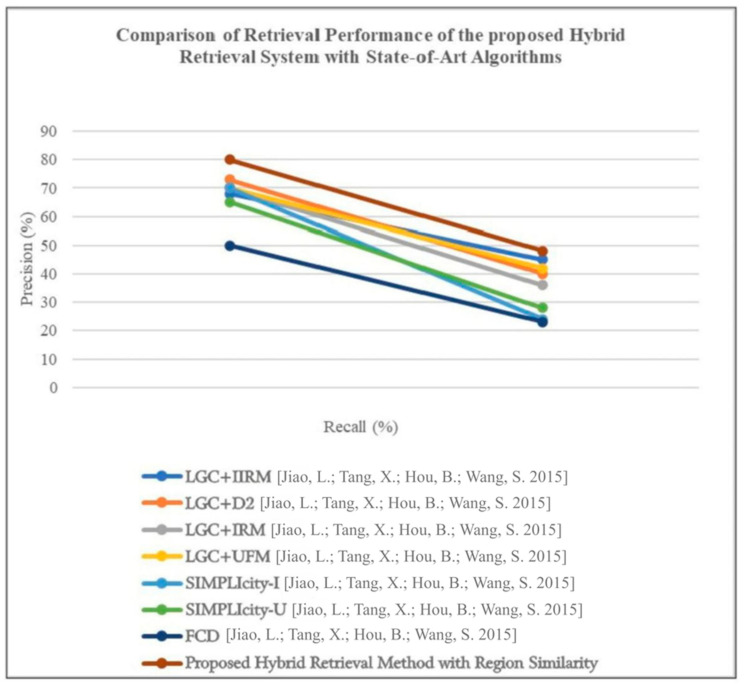
Comparison of the retrieval performance of the proposed hybrid retrieval method with the existing state-of-the-art algorithms [35].

To assess the efficacy of the proposed hybrid retrieval method in comparison to existing retrieval algorithms, a precision–recall graph is plotted as shown in Figure 4, evaluating various techniques on online datasets. The compared retrieval techniques include the FCD method [Jiao, L.; Tang, X.; Hou, B.; Wang, S., 2015] [35], which calculates similarity using the fast compression distance algorithm; SIMPLIcity-I [Jiao, L.; Tang, X.; Hou, B.; Wang, S., 2015] [35], classifying image patches into semantic groups and computing similarity using the IRM distance measure; SIMPLIcity-U [Jiao, L.; Tang, X.; Hou, B.; Wang, S., 2015] [35], employing the UFM distance measure; and Local Gradient Coding (LGC) with different distance measures, such as IRM, UFM, and D2.

In the evaluation, a dataset comprising 1500 samples from diverse categories like Mountain, Ocean, Port, High-density residential, Medium-density residential, Low-density residential, Farm, Plant, Mixed Forest, and Water bodies is employed. The precision in percentage vs. recall in percentage is plotted for different retrieval algorithms. Notably, the proposed hybrid retrieval method consistently outperforms the seven existing techniques in terms of precision and recall values.

LGC+D2 exhibits better performance, although its effectiveness diminishes with an increasing number of samples, leading to higher computational complexity. The proposed method, leveraging high-level features derived from CNN and low-level features from the modified MSMA-CLBP algorithm, significantly enhances classification accuracy compared to existing methods. Additionally, region-based global mapping is employed, and similarity is computed between query and target patches.

The proposed system demonstrates stable performance and low computational complexity, particularly when utilizing GPU capabilities. Across various categories, the proposed system exhibits improvements over existing techniques, with a dedicated focus on HSI/SAR images due to their intricate nature. Incorporating soft thresholding enhances post-classification accuracy, resulting in an overall improvement of 0.08%. 

#### Comparison with State-of-the-Art Algorithms

A. To evaluate the effectiveness of the proposed hybrid system, extensive comparisons were conducted against several state-of-the-art traditional and deep-learning-based methods across three critical modules: sample selection, image registration, and classification/retrieval. In the sample selection phase, conventional methods like the Bootstrap Transfer algorithm combined with SIFT achieved an accuracy of 63.71%. In contrast, the proposed method, MSMA-MSBT, which integrates multiscale feature fusion with active learning for unlabeled data selection, demonstrated a significant improvement with an accuracy of approximately 83.36%. This ~20% gain highlights the advantages of employing multiscale and multiangle image features along with intelligent sample selection, reducing manual labeling effort while enhancing representational richness. In the image registration module, the proposed Deep IRDI algorithm also proved superior. While traditional RANSAC-based methods yielded an accuracy of 84.14% and SIFT achieved only 71.71%, the proposed method reached a remarkable accuracy of 98.65%. Even advanced registration algorithms like the baseline IR model achieved 95.65%, falling short of the dynamic inlier strategy employed in Deep IRDI. This dynamic approach effectively adapts to local feature variations and overcomes the rigid constraints of fixed inlier definitions used in other methods, resulting in more accurate and consistent registration across multitemporal and multisource remote sensing images.

B. In the classification and retrieval stage, traditional approaches such as Bag of Visual Words, Locality-constrained Linear Coding, and Spatial Pyramid Matching using SIFT performed suboptimally, with classification accuracies typically below or around 80%. Deep learning models such as LPCNN have shown promising improvements, reaching classification accuracies close to 89.88%. However, the proposed hybrid approach, which combines MSMA-CLBP handcrafted features with high-level CNN-based features in a purpose-built architecture, surpassed these models with a classification accuracy of 92.25%. The improvement stems from the synergy of low-level texture descriptors and high-level abstract features learned by the CNN, allowing the system to better capture complex spatial and spectral patterns present in remote sensing data.

C. These comparative results clearly demonstrate that the proposed hybrid system consistently outperforms existing state-of-the-art methods across the board. Its modular architecture enables each component—whether sample selection, registration, or classification—to utilize the most effective techniques tailored to remote sensing imagery, addressing specific limitations of individual approaches. By intelligently integrating handcrafted and learned features, leveraging active learning strategies, and implementing dynamic registration techniques, the system delivers improved accuracy, robustness, and adaptability, thereby setting a new benchmark for land use and land cover mapping in remote sensing.

## 5. Conclusions

Increasing technological advancements in the field of remote sensing have made it easier to capture objects located at remote locations through satellites and aerial platforms. This has expanded the scope of numerous applications across environmental monitoring, urban planning, agriculture, and more. However, processing such complex imagery introduces significant technical challenges. Remote sensing images, particularly hyperspectral and synthetic aperture radar data, differ from traditional computer vision images in terms of spectral richness, noise characteristics, and spatial complexity.

With the increasing availability of large-scale remote sensing datasets, researchers are actively engaged in acquiring, storing, processing, and analyzing such data. However, despite the advancements in data collection, many systems still fall short in efficient manipulation, indexing, sorting, filtering, summarizing, or searching through such vast databases.

Content-based image retrieval techniques aim to address this by retrieving images based on their visual content, extracting features such as color, texture, and shape. However, content-based image retrieval systems face the “semantic gap” challenge—a disconnect between low-level features and high-level semantic concepts. This challenge is further amplified in remote sensing images due to mixed pixels, indistinct boundaries, and complex spatial patterns.

To address these issues, the present work proposes a comprehensive hybrid retrieval system. This system integrates multiple modules, starting from the preprocessing of HSI and SAR images, followed by dynamic inlier-based image registration, active learning-based sample selection using a proposed MSMA-MSBT technique, deep-learning-based classification, error correction via soft thresholding, and finally, a retrieval module based on region-based similarity matching.

The performance of this system is validated on both offline and online datasets, including the widely recognized AID dataset, which consists of 10,000 images across 30 classes. The focus is particularly on HSI/SAR images due to their richer information content compared to panchromatic or multispectral imagery.

### 5.1. Sample Selection Using Proposed Modified MSMA-MSBT

Classifier performance is significantly influenced by the selection of training samples. The proposed MSMA-MSBT approach selects informative unlabeled samples that enhance the training set quality. HSI/SAR images are augmented at multiple scales and rotated to create a fused dataset, improving performance over traditional single-scale methods.

Experiments show that the fused dataset achieves higher accuracy, with the proposed MSMA-MSBT algorithm yielding a classification accuracy of approximately 83.36%, compared to 63.71% using the standard BT-SIFT combination—a nearly 20% improvement. This demonstrates the effectiveness of multiscale augmentation and selective sampling in boosting classifier performance.

### 5.2. Image Registration Using Proposed Modified DeepIRDI

Image registration is crucial for HSI/SAR images, which are often captured at different times with varying resolutions and viewing angles. Traditional registration techniques using static inlier–outlier selection often fail in the presence of high variability.

The proposed modified DeepIRDI method adopts a dynamic inlier selection approach that reduces misregistration and enhances alignment. The proposed method achieves registration accuracy as high as 98.65% for satellite images and 95.43% for UAV images, significantly outperforming traditional techniques such as RANSAC, IR, and SIFT, whose accuracies range from 42% to 95%. The improvement is attributed to the adaptive and iterative inlier enhancement strategy.

### 5.3. Classification and Retrieval via Hybrid MSMA-CLBP-CNN

Image classification forms the foundation of retrieval accuracy. The hybrid classification approach integrates low-level features extracted via the modified MSMA-CLBP technique and high-level features obtained from a CNN architecture. These features are fused and classified using a softmax layer.

Additionally, a post-classification soft thresholding mechanism corrects misclassified pixels, particularly useful in mixed-pixel scenarios typical in HSI/SAR imagery. Retrieval is performed through a region-based patch similarity mechanism using the L2 norm, which identifies matching image regions with enhanced precision.

This approach achieves a classification accuracy of 92.25%, outperforming traditional methods like BoVW (73.93%) and LLC (70.89%), and even recent deep learning models such as LPCNN (89.88%). The hybrid strategy benefits from a dedicated CNN built from scratch tailored to the domain-specific characteristics of remote sensing imagery.

**Future Scope** **:**

***More* Features *can be Tested***

The feature selection process is crucial in the proposed hybrid retrieval system. While three features are currently considered, evaluating the system’s performance with a broader array of features could provide valuable insights.

***Application Building for Constrained Environment***

The suggested hybrid retrieval system has the potential for optimization and adaptation to a constrained environment. It could be reengineered to create a mobile application focused on effective online retrieval of HSI/SAR images, with the potential to support various other applications.

## Figures and Tables

**Table 1:** Selected samples using MSMA-MSBT algorithm from AID dataset.

Label	Count (Selected Samples/ Total Number of Samples)
Airport	287/360
Base Land	228/310
Baseball Field	170/220
Beach	293/400
Bridge	209/360
Centre	166/260
Church	180/240
Commercial	200/350
Dense Residential	349/410
Desert	158/300
Farmland	273/370
Forest	153/250
Industrial	293/390
Meadow	183/280
Medium Residential	193/290
Mountain	243/340
Park	253/350
Parking	293/390
Playground	273/370
Pond	323/420
Port	283/380
Railway Station	163/260
Resort	193/290
River	313/410
School	203/300
Sparse Residential	203/300
Square	233/330
Stadium	193/290
Storage Tanks	263/360
Viaduct	323/420

**Table 2:** Comparison of selected samples for training using the proposed MSMA-MSBT algorithm for HSI/SAR datasets.

Dataset	Proposed MSMA-MSBT Accuracy (%)	SIFT Accuracy (%) [6]
Satellite	84.14	71.71
UAV	79.25	42.94

**Table 3:** Comparison of proposed modified DeepIRDI with the existing state-of-the-art algorithms.

Dataset	Proposed Modified DeepIRDI	RANSAC [15]	IR [18]	SIFT [19]
Satellite	98.65%	84.14%	95.65%	71.71%
UAV	95.43%	79.25%	93.37%	42.94%

**Table 4:** Represents the comparison of proposed modified DeepIRDI with the RANSAC algorithm with respect to angle, resolution, and fusion dataset.

Dataset	Parameter	Modified Deep IRDI (Proposed)	RANSAC [15]
Satellite dataset with angle variant Dataset A	Avg	87.64	83.30
	Min	77.25	82.57
	Max	98.65	84.71
Satellite dataset with resolution variant Dataset B	Avg	82.75	80.54
	Min	81.00	76.19
	Max	84.14	85.10
Satellite dataset with Angle and resolution Fusion Dataset C	Avg	86.38	81.98
	Min	84.49	73.83
	Max	88.99	90.81

**Table 5:** Comparison of SAR/hyperspectral images using various pretrained networks.

Accuracy	Original Image	Features of Original Image	Multiscaled Image	Features of Multiscaled Image
Alexnet	83.213	70.504	98.321	75.540
Caffenet	78.657	79.616	98.321	82.254
VGG-F	87.530	78.177	96.643	79.616
VGG-M	87.290	69.305	98.801	70.743
VGG-S	88.010	73.621	99.041	78.657
VGG-VDD-16	76.499	71.463	97.602	75.779
VGG-VDD-19	78.897	72.902	98.321	73.861

**Table 6:** Comparison of GPU and CPU training time in seconds of SAR/hyperspectral images using various pretrained networks.

	Training Time per Epoch (Seconds) with CPU	Training Time per Epoch (Seconds) with GPU
Alexnet	312.000	156.00
Caffenet	315.000	157.500
VGG-F	702.000	351.000
VGG-M	798.000	399.000
VGG-S	815.000	407.500
VGG-VDD-16	1500.000	750.000
VGG-VDD-19	1800.000	900.000

**Table 7:** Comparison of the proposed hybrid modified MSMA-CLBP-CNN with existing state-of-the-art algorithms for online datasets (highest accuracy reported).

Parameters	Classification Method	Classification Accuracy (%)
Traditional Methods	Bag of Visual Words (BoVW) [21]	73.93
	Spatial Pyramid Matching using SIFT (SPM-SIFT) [21]	80.26
	Locality-constrained Linear Coding (LLC) [21]	70.89
	LDA [21]	66.85
Deep Learning Methods	S-UFL [22]	74.84
	LPCNN [23]	89.88
	Hybrid modified MSMA-CLBP-CNN (Proposed)	92.25

**Table 8:** Comparison of the proposed multiscale pretrained network classification with existing state-of-the-art algorithms for online datasets.

Convolutional Neural Networks	Classification Accuracy %
Spatial BOW [27]	81.19
SIFT + BOW [28]	75.11
SC + Pooling [29]	81.67
SPM [30]	86.8
PSR [31]	89.1
Caffenet [12]	95.02
Google net [32]	94.31
GBRCN [33]	94.53
Caffenet + fine tuning [12]	95.48
Google net + fine tuning [32]	97.1
Multiscale + Alexnet [Proposed Multiscale Algorithm]	98.32
Multiscale + Caffenet [Proposed Multiscale Algorithm]	98.32
Multiscale + VGG-F [Proposed Multiscale Algorithm]	96.64
Multiscale + VGG-M [Proposed Multiscale Algorithm]	98.8
Multiscale +VGG-S [Proposed Multiscale Algorithm]	99.04
Multiscale + VGG-VDD-16 [Proposed Multiscale Algorithm]	78.9
Multiscale + VGG-VDD-19 [Proposed Multiscale Algorithm]	98.32

**Table 9:** Quantitative and qualitative parametric values for offline datasets with proposed hybrid architecture for HSI/SAR image retrieval.

Dataset	Entropy	Energy	Co-Relation	Queue	PSNR	MSE	RSME	Quality Index	Kappa	SNR	MAE
0°	6.87	2.08	0.72	0.32	15.67	11.01	4.00	0.31	1.00	7.18	7.26
90°	7.38	2.57	0.77	0.35	15.12	11.57	4.01	0.38	0.97	7.30	7.50
180°	6.99	2.59	0.75	0.32	15.99	11.46	4.06	0.39	1.00	7.24	7.31
270°	6.79	2.04	0.78	0.33	15.79	11.69	4.07	0.41	1.00	7.36	7.44
64 × 64	7.25	3.00	0.79	0.40	15.88	12.00	4.19	0.44	0.80	7.87	8.47
256 × 256	6.90	2.22	0.73	0.34	15.37	11.35	4.20	0.44	1.00	7.11	7.27
512 × 512	7.13	2.20	0.78	0.30	15.92	11.47	4.23	0.51	1.00	7.26	7.39
90°-64 × 64	7.11	3.00	0.73	0.40	16.02	11.97	4.30	0.60	0.75	7.19	7.06
90°-256 × 256	6.81	2.42	0.74	0.38	15.99	11.87	4.40	0.60	0.74	7.27	7.31
90°-512 × 512	7.36	2.92	0.80	0.35	16.00	11.57	4.41	0.62	1.00	7.22	7.61
180°-64 × 64	7.59	2.99	0.80	0.38	15.76	12.00	4.48	0.67	0.82	7.60	7.87
180°-256 × 256	6.90	2.13	0.79	0.39	16.00	11.99	4.50	0.70	1.00	7.17	7.25
180°-512 × 512	7.46	3.02	0.73	0.31	15.79	11.67	4.49	0.70	1.00	7.24	7.41
270°- 64 x64	7.21	2.99	0.76	0.40	16.00	12.00	4.42	0.69	0.91	7.45	7.67
270°-256 × 256	7.15	2.72	0.71	0.32	15.68	11.35	4.50	0.70	1.00	7.26	7.26
270°-512 × 512	7.65	2.94	0.80	0.32	16.00	11.54	4.49	0.70	1.00	7.25	7.39

**Table 10:** Quantitative and qualitative parametric values for online AID dataset with proposed hybrid architecture for HSI/SAR image retrieval.

Datasets	Entropy	Energy	Co-Relation	PSNR	MSE	RMSE	Quality Index	Kappa	SNR	MAE
Airport	7.15	3.52	0.99	18.24	9.82	3.13	0.27	0.64	5.86	6.46
Base Land	6.50	2.30	1.00	18.91	8.42	2.90	0.17	0.92	5.19	6.39
Baseball Field	7.15	3.52	1.00	18.24	9.82	3.13	0.27	0.64	5.85	6.46
Beach	7.09	2.79	0.99	18.54	9.17	3.03	0.16	0.64	5.64	6.33
Bridge	7.15	3.52	0.99	18.24	9.81	3.13	0.27	0.64	5.85	6.46
Centre	7.15	3.52	0.99	18.24	9.82	3.13	0.27	0.64	5.85	6.46
Church	7.15	3.52	0.99	18.24	9.82	3.13	0.27	0.64	5.85	6.46
Commercial	7.15	3.52	0.99	18.24	9.82	3.13	0.27	0.64	5.85	6.46
Dense Residential	7.15	3.52	1.00	18.24	9.82	3.13	0.27	0.64	5.85	6.46
Desert	4.30	5.45	0.98	17.72	11.06	3.33	3.33	0.93	6.37	6.34
Farmland	5.13	3.97	0.96	17.79	10.89	3.29	0.15	0.92	6.30	6.36
Forest	6.87	1.75	0.99	18.54	9.16	3.03	0.34	0.64	5.56	6.30
Industrial	7.12	1.86	0.99	18.70	8.83	2.97	0.29	0.91	5.39	6.33
Meadow	4.67	7.51	0.88	18.38	9.52	3.08	0.09	0.95	5.72	6.39
Medium Residential	6.78	6.31	1.00	17.92	10.58	3.25	0.25	0.98	6.35	6.36
Mountain	5.86	3.56	0.96	17.64	11.29	3.36	0.26	0.96	6.46	6.41
Park	7.04	2.18	0.98	18.73	8.78	2.96	0.39	0.93	5.37	6.34
Parking	6.37	1.24	0.99	18.52	9.22	3.04	0.32	0.92	5.58	6.37
Playground	6.80	7.55	1.00	18.04	10.29	3.21	0.21	0.91	6.09	6.37
Pond	6.61	1.27	0.99	18.53	18.53	3.03	0.27	0.90	5.57	6.37
Port	6.59	8.89	1.00	17.88	10.68	3.27	0.16	0.59	6.22	6.45
Railway Station	6.55	1.99	0.97	18.90	8.45	2.91	0.30	0.95	5.20	6.36
Resort	7.57	2.00	0.97	18.32	9.64	3.11	0.39	0.52	5.78	6.39
River	7.15	3.52	1.00	18.24	9.82	3.13	0.27	0.64	5.85	6.46
School	7.04	1.32	0.98	18.09	10.17	3.19	0.45	0.52	6.01	6.36
Sparse Residential	6.39	1.29	0.92	18.69	8.86	2.98	0.29	0.99	5.42	6.39
Square	7.23	3.96	1.00	17.98	10.44	3.23	0.22	0.39	6.12	6.32
Stadium	7.15	3.52	1.00	18.24	9.82	3.13	0.27	0.64	5.85	6.46
Storage Tanks	7.26	2.18	0.99	18.45	9.38	3.06	0.38	0.61	5.66	6.40
Viaduct	6.73	9.33	0.99	18.28	9.74	3.12	0.32	0.96	5.84	6.34

**Table 11:** Comparison of average existing precision value with average precision value by proposed method.

Datasets	Average Existing Precision Value [35]	Average Precision Value Using Proposed Hybrid Retrieval Algorithm Based on Region Similarity
AID	0.33	0.79
UC_MERCED	0.51	0.83
WHU_RS	0.66	0.83

**Table 12:** Comparison of average existing recall value with average recall value by proposed method.

Datasets	Average Existing Recall Value [35]	Average Recall Value Using Proposed Hybrid Retrieval Algorithm Based on Region Similarity
AID	0.49	0.78
UC_MERCED	0.57	0.82
WHU_RS	0.64	0.82

**Table 13:** Comparison of average existing F1 score value with average F1 score value by proposed method.

Datasets	Average Existing F1 Score Value [35]	Average F1 Score Value Using Proposed Hybrid Retrieval Algorithm Based on Region Similarity
AID	0.37	0.79
UC_MERCED	0.51	0.82
WHU_RS	0.64	0.82

## Data Availability

The offline dataset is prepared by the techniques described in the manuscript whereas the online dataset is taken from AID:A Benchmark Dataset for Performance Evaluation of Aerial Scene Classification, UC MERCED: http://weegee.vision.ucmerced.edu/datasets/landuse.html (accessed on 1 July 2023), WHU_RS: https://drive.google.com/drive/folders/16a9HRnhd2EcM_4CnCFpQL1xCJFUb6VA6?usp=sharing (accessed on 16 October 2023).
